# Comparative Analysis of Two Soybean Cultivars Revealed Tolerance Mechanisms Underlying Soybean Adaptation to Flooding

**DOI:** 10.3390/cimb46110739

**Published:** 2024-11-04

**Authors:** Xiaobo Yu, Jiangang An, Jianqiu Liang, Wenying Yang, Zhaoqiong Zeng, Mingrong Zhang, Haiying Wu, Sichen Liu, Xiaoning Cao

**Affiliations:** 1 Nanchong Academy of Agricultural Sciences, Nanchong 637000, China; bo0524@163.com (X.Y.); ajgang0605@163.com (J.A.); liangjianqiu142@163.com (J.L.); yangwenying1315@163.com (W.Y.); 13696028982@163.com (Z.Z.); 13890812316@163.com (M.Z.); why2800531@163.com (H.W.); 2 Sweetpotato and Leguminosae Germplasm Innovation and Utilization Key Laboratory of Sichuan Province, Nanchong 637000, China; 3 Center for Agricultural Genetic Resources Research, Shanxi Agricultural University, Taiyuan 030031, China

**Keywords:** soybean, flooding stress, *MET1*, *DME*, abiotic stress adaptation

## Abstract

Flooding stress poses a significant challenge to soybean cultivation, impacting plant growth, development, and ultimately yield. In this study, we investigated the responses of two distinct soybean cultivars: flooding-tolerant Nanxiadou 38 (ND38) and flooding-sensitive Nanxiadou 45 (ND45). To achieve this, healthy seedlings were cultivated with the water surface consistently maintained at 5 cm above the soil surface. Our objective was to elucidate the physiological and molecular adaptations of the two cultivars. Under flooding stress, seedlings of both cultivars exhibited significant dwarfing and a notable decrease in root length. While there were no significant differences in the dry weight of aboveground shoots, the dry weight of underground shoots in ND38 was strikingly decreased following flooding. Additionally, total chlorophyll content decreased significantly following flooding stress, indicating impaired photosynthetic performance of the cultivars. Moreover, malondialdehyde (MDA) levels increased significantly after flooding, particularly in the ND45 cultivar, suggesting heightened oxidative stress. Expression analysis of methylation and demethylation genes indicated that *MET1* and *DME* play crucial roles in response to flooding stress in soybeans. Meanwhile, analysis of the hemoglobin family (GLBs), aquaporin family (AQPs), glycolytic pathway-related genes, and NAC transcription factor-related genes identified *GLB1-1* and *GLB1-2*, *GLB2-2*, *PIP2-6*, *PIP2-7*, *TIP2-2*, *TIP4-1*, *TIP5-1*, *Gm02G222400* (fructose-bisphosphate aldolase), *Gm19G017200* (glucose-6-phosphate isomerase), and *Gm04G213900* (alcohol dehydrogenase 1) as key contributors to flooding tolerance in both soybean cultivars. These findings provide crucial insights into the physiological and molecular mechanisms underlying flooding tolerance in soybeans, which could guide future molecular breeding strategies for the development of flooding-tolerant soybean cultivars.

## 1. Introduction

Soybeans (*Glycine max*) are one of the world’s most important legume crops, widely cultivated for their high nutritional value, particularly as a rich source of high-quality protein and essential fatty acids [1]. They play crucial roles in the agronomic and food sectors, contributing significantly to global food security and livestock feed [2]. The yield and quality of soybeans are determined by multiple factors, including organ size regulation, plant architecture, and stress tolerance, which together play crucial roles in shaping the growth, development, and overall productivity of soybean plants [3,4,5]. Furthermore, environmental factors such as temperature [6], water availability conditions [7], illumination [8], and nutrient levels [9] also influence the yield of soybeans [10,11,12]. Additionally, soybean cultivation is increasingly challenged by altered temperature and precipitation patterns, more frequent extreme weather events, water stress, and increased pest and disease pressures [13]. To address these challenges, multiple measures have been developed to boost soybean tolerance, such as improvements in alkali tolerance [14], salt tolerance [15], drought tolerance [16], and flooding tolerance [17].

Flooding is a major abiotic stress that significantly affects soybean cultivation, leading to substantial yield losses [18]. Soybean represents one of the key grain crops in China [19]. Historically, China frequently experiences seasonal flooding, particularly in regions such as the Yellow River Basin and the Yangtze River Basin, mostly during the hot and rainy summer months [20,21]. These flooding conditions underscore the necessity for flood-tolerant cultivars and effective management strategies to enhance crop resilience in this critical agricultural zone. Flooding stress impacts the entire developmental stage of soybean plants, including seed germination, vegetative growth, and reproductive growth. It disrupts normal physiological and metabolic processes, causing structural damage, impaired root function, chlorosis, and plant death [18,22]. Young plants are particularly vulnerable during critical developmental stages, such as germination and early seedling growth [23,24]. Research indicates that these stages are critical, as seedlings often lack established root systems to anchor them in saturated soils [25].

Plants have evolved complex responses to flooding stress, involving various signaling pathways, gene regulation, and biochemical adaptations. Antioxidant enzymes, like superoxide dismutase (SOD) and peroxidase (POD), mitigate oxidative stress by scavenging reactive oxygen species (ROS) generated during hypoxic conditions [26]. Additionally, hemoglobin genes in species such as *Arabidopsis*, tomato, and soybean play a crucial role in reducing nitric oxide (NO) accumulation, which helps maintain cellular homeostasis under low-oxygen environments [27,28,29,30]. Aquaporin genes, involved in water transport across cell membranes, have also been identified as critical regulators of flooding responses in several plant species, including sorghum [31] and trifoliate orange [32], enhancing cellular water balance under stress.

Flooding stress also triggers metabolic changes, particularly in pathways related to carbohydrate metabolism [33,34]. Peanuts experiencing flooding have been found to exhibit disruptions in starch and sucrose metabolism within their leaves [35]. Glycolysis/gluconeogenesis serves as a primary energy source under hypoxic conditions, with enzymes such as alcohol dehydrogenase (ADH) facilitating anaerobic respiration to sustain energy production. In glycolysis/gluconeogenesis, the gene encoding alcohol dehydrogenase (ADH) promotes alcohol fermentation, thereby providing NAD+ to maintain the glycolytic pathway. Under flooding stress, some genes are significantly overexpressed, underscoring the importance of the ADH-dependent glycolytic pathway in enhancing plant tolerance to flooding. Furthermore, transcription factors (TFs) like MYB, AP2, NAC, and WRKY are key regulators that activate stress-responsive genes, contributing to adaptive responses under waterlogged conditions [36,37,38,39]. Furthermore, methylation and demethylation pathways are critical in regulating gene expression in response to flooding, underscoring the role of epigenetic modifications in stress-tolerance mechanisms. The methylation and demethylation of various signaling molecules contribute to enhancing the ability to withstand flooding stress in plants [40,41]. The transcript levels of methylated and demethylated signals are further affected by flooding [42]. In wheat, genes such as *ERF1*, *ACC1*, and *CKX2.3* have been identified as flooding-related genes, with their expression prominently regulated by demethylation processes [41].

While general responses to flooding have been well-documented, the underlying chemical and molecular mechanisms that enable plants to tolerate flooding remain incompletely understood. Despite its extensive cultivation, soybean remains highly susceptible to flooding stress, highlighting an urgent need for the development of flood-tolerant cultivars. Therefore, urgent efforts are needed to breed high-quality soybean germplasm to safeguard food security and optimize yields. In this study, we conducted flooding treatment on two soybean cultivars exhibiting varying degrees of tolerance to flooding. By exploring their physiological and molecular disparities, our aim was to uncover the underlying mechanisms driving their distinct responses to flooding stress. This study provides a foundation for breeding strategies that enhance soybean resilience to this increasingly prevalent environmental challenge.

## 2. Materials and Methods

### 2.1. Plant Materials and Flooding Treatment

The seeds of two soybean cultivars, flooding-tolerant Nanxiadou 38 (ND38) and flooding-sensitive Nanxiadou 45 (ND45), were provided by the Nanchong Academy of Agricultural Sciences. These two cultivars were planted in July in the Center for Agricultural Genetic Resources Research, Shanxi Agricultural University. After five days of seed germination, 40 uniform and healthy seedlings were randomly selected from each cultivar based on visible seedling viability and growth consistency, such as similar size, leaf development, and absence of any visible abnormalities or damage. These seedlings were then divided into two groups: the flooding group and the control group. In the flooding group, seedlings were subjected to flooding treatment, with the water surface always maintained at 5 cm above the soil surface. The control group underwent regular irrigation once a day in the afternoon. The average temperature for the experiment under natural conditions was 25 °C during the day and 16 °C at night. The matrix ratio used for planting was peat/vermiculite/perlite = 5:2:1. After 10 days of flooding treatment, the entire plants, including roots, were harvested for further analysis.

### 2.2. Determination of Chlorophyll Content

The first and second young leaves, except the apical bud, were cut from each seedling for chlorophyll measurement. Subsequently, 1 g of the leaf sample was added with 14 mL of 96% ethanol for extraction. After incubation for 3 days at 60 °C, the absorbance values at wavelengths of 645 nm, 652 nm, and 663 nm were measured, enabling the calculation of chlorophyll content.

### 2.3. Chlorophyll Fluorescence Imaging

Chlorophyll fluorescence images of leaf samples were obtained by a multispectral phenotyping platform (TraitDiscover, PhenoTrait, Beijing, China). This platform allows visualization of various physiological traits, based on specific absorption, reflection, and emission spectra. Chlorophyll index (ChlIdx), photosystem II efficiency (Fv/Fm), and anthocyanin reflection index (AriIdx) were measured, and due imaging was captured following manufacturer’s instructions.

### 2.4. Biochemical Indicators

An appropriate amount of the clean root sample from soybean plants was ground thoroughly after adding with liquid nitrogen. The obtained tissue powder was divided into three portions: two were homogenized in PBS (tissue weight (g):PBS (mL) volume = 1:9) for malondialdehyde (MDA) and peroxidase (POD) tests, respectively, while one portion was homogenized in double-distilled water (tissue weight (g):PBS (mL) volume = 1:9) for an SOD test. After sample preparation, MDA assay kits (TBA method), peroxidase assay kits, and SOD assay kits (WST-1 method), purchased from Nanjing Jiancheng Biotechnology Research Institute (Nanjing, Jiangsu, China), were used for MDA, POD, and SOD determination.

### 2.5. RNA Extraction and Quantitative Real-Time Polymerase Chain Reaction (qRT-PCR)

The Trizol total RNA extraction kit (Tiangen Biotech, Beijing, China) was used to extract RNA from 100 mg of root sample according to manufacturer’s instructions. RNA extraction quality was assessed using a UV spectrometer (NanoDrop, ThermoFisher, Waltham, MA, USA). RNA was reverse transcribed into cDNA through the HiScript III 1st Strand cDNA Synthesis Kit (Vazyme, Nanjing, Jiangsu, China). Primers were designed using the Primer-BLAST tool on NCBI (https://www.ncbi.nlm.nih.gov/tools/primer-blast/, accessed on 7 August 2023). qRT-PCR reaction was performed on a TaqMan Fast Advanced Master Mix (ThermoFisher, MA, USA). The cycling conditions were 95 °C pre−denaturation for 10 min, 95 °C denaturation for 15 s, and 60 °C annealing for 1 min. qRT-PCR analysis was conducted on the Applied Biosystems 7500 (ThermoFisher, Waltham, MA, USA). qRT-PCR analysis included three biological replicates for each sample to ensure reliability and robustness. Quantitative data were calculated by the 2^−ΔΔCt^ method. Pairwise comparisons were conducted using Student’s *t*-test. *ACT11* was used as the internal standard. Primer sequences, as well as corresponding references for each gene in soybean, are presented in Table 1.

### 2.6. Statistical Analysis

All statistical analyses were conducted using two-tailed Student’s *t*-tests, with significance thresholds set at * *p* ≤ 0.05, ** *p* ≤ 0.01, and **** *p* ≤ 0.001. Data visualization was performed using the ggplot2 package in R Studio 4.3.1, while the ggsignif package was utilized to perform significance testing (*p* < 0.05). Results are expressed as mean ± standard error (SE), with error bars included to illustrate data variability in bar plots.

## 3. Results

### 3.1. Flooding Treatment Influenced the Normal Growth of Soybean Plants from Both Cultivars

**Figure 1:**
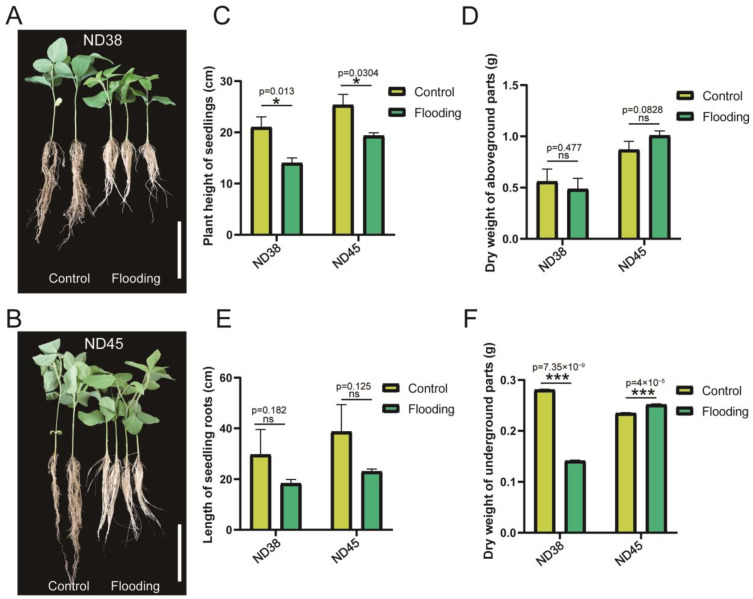
Impacts of flooding on the growth of soybean plants from two cultivars: Nanxiadou38 (ND38) and Nanxiadou45 (ND45). (**A**,**B**) Soybean seedlings demonstrated the appearance of dwarf forms under flooding stress. (**C**,**D**) Seedling height (**C**) and root length (**D**) showed distinct differences after flooding compared with the control group. (**E**,**F**) The comparison of aboveground (**E**) and underground (**F**) dry weight between flooding and control groups. * *p* ≤ 0.05, *** *p* ≤ 0.001 (Student’s *t*-test).

In order to evaluate the impact of flooding on soybean growth during the vegetative period, we collected the growth parameters of soybean seedlings from the treatment and control groups (Table S1). Both cultivars were able to grow under flooding stress, but they displayed distinct responses in the aboveground and underground parts. The seedlings of ND38 and ND45 exhibited significant dwarfing, with a notable reduction in root length under flooding stress compared with the control group (Figure 1A–D). In addition, we also measured the dry weight of aboveground and underground shoots. Neither cultivars showed a significant difference in the dry weight of aboveground parts, while ND38 demonstrated a striking decrease in underground dry weight when subjected to flooding (Figure 1E,F). These results indicated that the normal growth of soybean plants was disrupted under flooding stress.

### 3.2. Flooding Treatment Affected Chlorophyll Metabolism

**Figure 2:**
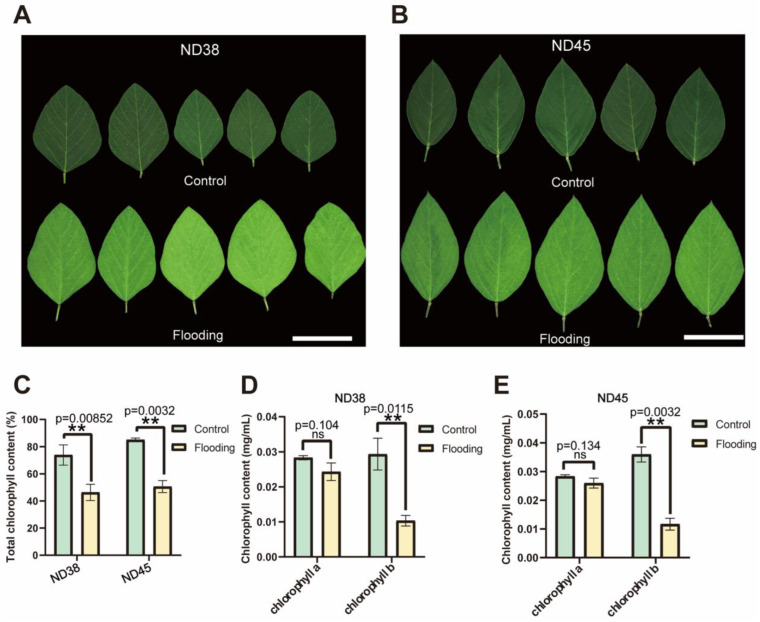
Analyses of chlorophyll content in soybean leaves under flooding conditions. (**A**,**B**) Flooded and normal leaves of ND38 (**A**) and ND45 (**B**). (**C**–**E**) Determination of total chlorophyll (**C**), chlorophyll a and chlorophyll b content of ND38 (**D**) and ND45 (**E**) in flooded and normal leaves from both cultivars. ** *p* ≤ 0.01 (Student’s *t*-test). Error bars indicate the standard error of the mean (*n* = 3).

To assess the impact of flooding on chlorophyll metabolism in soybean plants, we conducted analyses of chlorophyll content in the leaves of flooding and control plants (Table S2). We noticed apparent leaf yellowing in flooded leaves compared with the control group (Figure 2A,B). A significant decrease was observed in the total chlorophyll content of both cultivars after flooding treatment, with chlorophyll b exhibiting the most pronounced reduction. In contrast, the content of chlorophyll a was not significantly changed (Figure 2C–E). We speculated that the decrease in total chlorophyll content may be attributed to changes in the content of chlorophyll b, and that chlorophyll b may be more susceptible to degradation or turnover under flooding stress compared with chlorophyll a. Further research is needed to fully understand the physiological implications of this specific response, and its consequences for plant growth and productivity under flooding conditions.

### 3.3. Flooding Treatment Impacted Photosynthetic Capacity in Soybean Leaves

**Figure 3:**
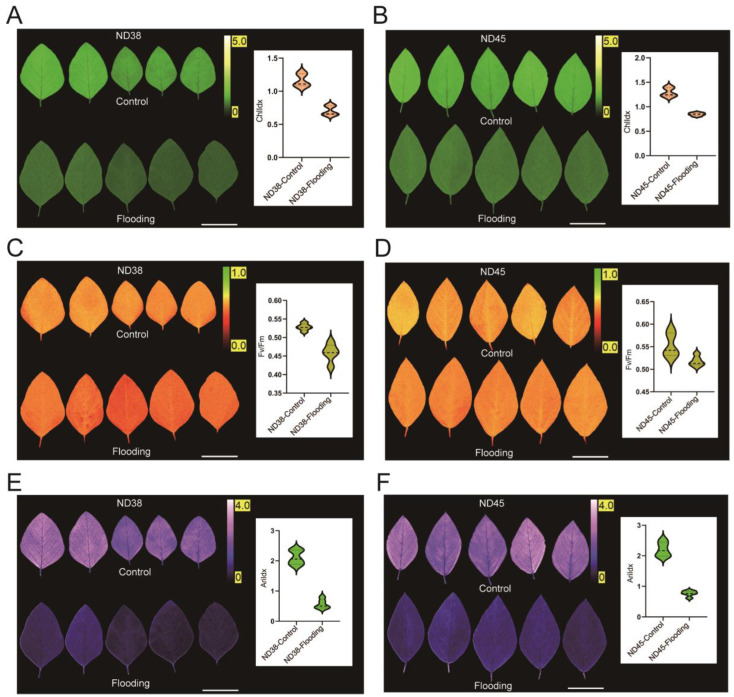
Measurement of leaf fluorescence indexes. (**A**,**B**) Chlorophyll index (ChlIdx) detection. (**C**,**D**) Fv/Fm index detection. (**E**,**F**) Anthocyanin index (AriIdx) detection.

To assess the impact of flooding treatment on the photosynthetic capacity and performance of soybean plants, changes in ChlIdx, Fv/Fm and AriIdx were analyzed. We observed a notable reduction in ChlIdx of both cultivars following flooding treatment (Figure 3A,B). Additionally, ND38 exhibited a greater reduction in Fv/Fm than ND45 (Figure 3C,D). Furthermore, in the measurement of anthocyanins, we found that AriIdx decreased nearly twofold in both cultivars compared to the control group (Figure 3E,F). Overall, flooding significantly affected the photosynthetic processes in both soybean cultivars.

### 3.4. Flooding Treatment Induced Changes in Key Oxidative Indicators

**Figure 4:**
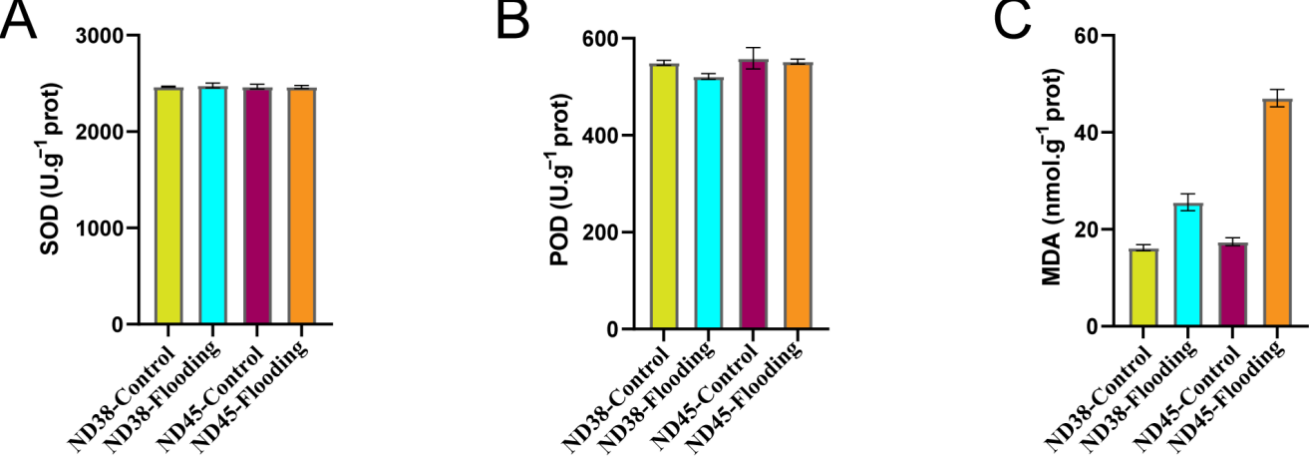
Changes in the levels of superoxide dismutase (SOD) (**A**), peroxidase (POD) (**B**), and malondialdehyde (MDA) (**C**) in soybean leaves. Error bars indicate the standard error of the mean (*n* = 3).

The levels of SOD, POD, and MDA were measured to evaluate the antioxidative response and oxidative damage induced by flooding treatment. Absorbance readings were taken at 450 nm, 420 nm, and 532 nm, respectively. SOD and POD, as key antioxidants, showed no significant differences in activity between the flooding and control groups for both cultivars (Figure 4A,B). However, MDA levels, an indicator of oxidative damage, displayed significant changes after flooding, particularly in the ND45 cultivar (Figure 4C). This suggests that ND45 may be more susceptible to oxidative damage under flooding conditions compared to ND38, highlighting differences in their antioxidative defense mechanisms.

### 3.5. Flooding Treatment Altered Epigenetic Modifications in Soybean Plants

**Figure 5:**
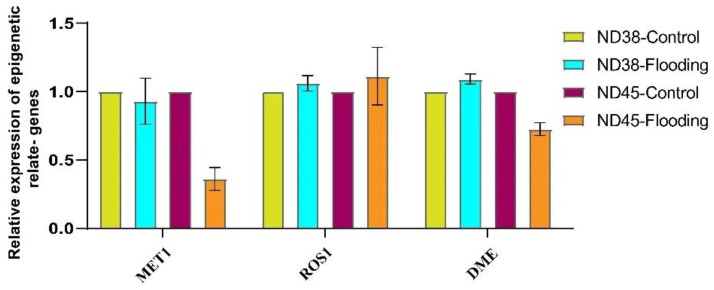
Impacts of flooding treatment on the expression of epigenetic-related genes. Error bars indicate the standard error of the mean (*n* = 3).

To investigate the relationship between flooding stress and epigenetic modification, we detected the expression changes of key methylation- (*MET1*) and demethylation-related (*ROS1* and *DME*) genes (Figure 5). The results showed a significant reduction in the expression of *MET1* following flooding treatment in ND45, whereas no significant difference was observed in ND38. Moreover, demethylation-related genes showed no difference in the expression of *ROS1* between the control and flooding groups in both cultivars. However, *DME* was significantly downregulated in the flooding group of ND45.

### 3.6. Flooding Treatment Affected the Expression of Environmental Adaptation Factors

**Figure 6:**
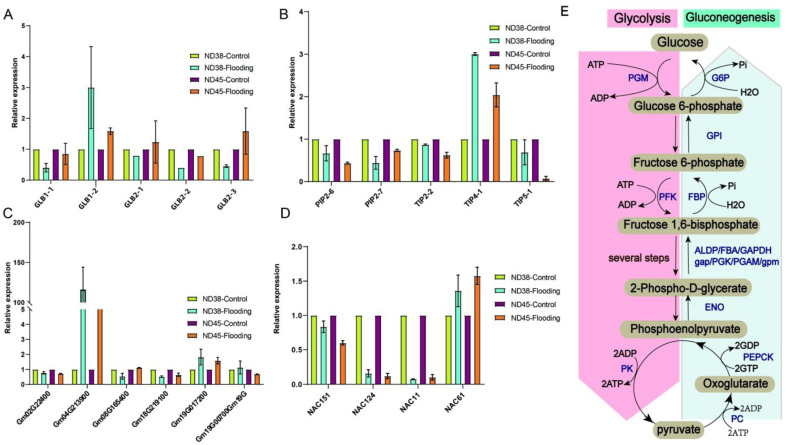
Alterations in the expression of environmental adaptation factors induced by flooding treatment. (**A**) Changes in the relative expression of hemoglobin genes. (**B**) Changes in the relative expression of aquaporin genes. (**C**) Changes in the relative expression of genes associated with the glycolysis/gluconeogenesis pathway. (**D**) Changes in the relative expression of NAC transcription factors (TFs). (**E**) Glycolysis/Gluconeogenesis pathway. *Gm02G222400*, fructose-bisphosphate aldolase; *Gm08G165400*, phosphoglycerate kinase; *Gm18G219100*, phosphoglycerate mutase (2,3-diphosphoglycerate-independent); *Gm04G213900*, alcohol dehydrogenase 1; *Gm19G017200*, glucose-6-phosphate isomerase; *Gm19G000700*, pyruvate kinase. Error bars indicate the standard error of the mean (*n* = 3).

In order to further understand the impact of flooding on plant adaptation to environmental stressors, we investigated the expression changes of several key environmental adaptation factors. We found a significant reduction in the expression of hemoglobin genes *GLB1-1*, *GLB2-2*, and *GLB 2-3* in the flooding group of ND38, which are known for their oxygen transport function. In the waterlogged group of ND45, the expression levels of *GLB1-2* and *GLB2-3* were upregulated compared with the control group (Figure 6A). As for aquaporin genes, the expression of *PIP2-6*, *PIP2-7*, *TIP2-2*, and *TIP5-1* were decreased in both ND38 and ND45 under flooding stress, but not *TIP4-1* (Figure 6B). Meanwhile, expression analysis of genes related to the glycolysis/gluconeogenesis pathway (Figure 6E) showed that *Gm02G222400* (fructose-bisphosphate aldolase), *Gm08G165400* (phosphoglycerate kinase), and *Gm18G219100* (phosphoglycerate mutase (2,3-diphosphoglycerate-independent)) were reduced in the flooding group of ND38, while *Gm04G213900* (alcohol dehydrogenase 1), *Gm19G017200* (glucose-6-phosphate isomerase) and *Gm19G000700* (pyruvate kinase) showed increased expression levels. In contrast, in ND45, the expression of *Gm02G222400*, *Gm18G219100*, and *Gm19G000700* was decreased, while that of *Gm04G213900*, *Gm08G165400*, and *Gm19G01720* increased compared with the control group. Among these genes, the expression patterns of *Gm02G222400*, *Gm18G219100*, and *Gm19G017200* were consistent across the two cultivars, indicating a potential conservation of these genes (Figure 6C). The results regarding NAC TFs indicated that, apart from NAC61, which showed upregulation, the expression levels of *NAC151*, *NAC124*, and *NAC11* were all downregulated under flooding stress in both cultivars. Notably, NAC124 and NAC11 exhibited the most significant decrease in expression levels compared with the other TFs (Figure 6D). These findings suggest that the expression patterns of identical genes remain largely consistent across the two cultivars, implying a probable functional conservation.

## 4. Discussion

By comparing the distinct responses of two soybean cultivars to flooding treatment, we collected relevant data on plant height, root length, dry matter weight, total chlorophyll content, and chlorophyll fluorescence index, as well as on molecular levels. The results further proved that ND38 had better flooding tolerance compared with ND45. In addition, multiple pathways, including methylation and demethylation, hemoglobin, aquaporins, glycolysis/gluconeogenesis pathways, and transcription factors, were involved in the response to flooding stress.

In plain and low-lying regions, flooding poses a recurrent natural hazard, especially during the rainy season. The limited flooding tolerance in most terrestrial plants frequently results in diminished crop yield and quality [50,51,52,53,54]. Recently, there has been a growing focus on flooding research aimed at cultivating high-quality crop cultivars. Significant breakthroughs have been made, and multiple genes associated with flooding tolerance have been identified. For example, the overexpression of *PhERF2*, *AtACO5*, *OsSK1/2*, *Sub1A*, and *ZmEREB180* enhances flooding tolerance in transgenic lines [37,55,56,57,58]. However, in soybeans, only a few genes have been reported, such as *GmAdh2*, *GmXTH*, and *GmNCED* [59,60,61]. Moreover, the precise molecular mechanisms underlying soybean flooding tolerance remain unclear, and the adoption of flooding-tolerant cultivars in agricultural production remains limited, despite widespread promotional efforts.

Flooding affects multiple physiological traits of plants, including seed germination, leaf senescence, stomatal closure, plant structure, photosynthetic efficiency, seed size, vegetative growth, and reproductive growth [62,63]. Sathi et al. indicated delayed flowering and maturity under flooding conditions [64]. Moreover, the symbiotic nitrogen fixation in nodules, vital for the growth and yield of leguminous crops, is also compromised by flooding. Collectively, these findings underscore the adverse effects of flooding on the optimal growth of plants. This study investigated the physiological responses of two soybean cultivars to flooding stress. It was found that both ND38 and ND45 experienced a decrease in plant height. Furthermore, ND38 exhibited a significant reduction in underground dry matter weight compared with the control group. In addition, the total chlorophyll content and photosynthetic pigment index declined in comparison to the control group. These results indicated that flooding may disrupt normal plant growth by affecting plant photosynthesis.

Investigating the molecular mechanisms governing soybean responses to flooding damage holds promise for the development of flooding-tolerant soybean varieties. Under flooding stress, plants encounter challenges related to oxygen transportation, water balance, and energy provision, necessitating adaptive mechanisms for survival. For instance, GLB proteins have been identified as facilitators of oxygen transport during water stress, thereby stimulating plant growth and lateral root elongation. Additionally, channel proteins like AQPs facilitate the movement of water and solutes across cell membranes, thereby sustaining normal cellular processes. Energy requirements are met through pathways such as glycolysis/gluconeogenesis and alcohol fermentation. These adaptive mechanisms underscore the dynamic strategies employed by plants to mitigate the impacts of flooding stress.

Epigenetic modifications play a crucial role in regulating plant growth and development, interacting with diverse environmental factors to either activate or inhibit the expression of tolerance genes. This enables plants to adapt to a wide range of challenging conditions [65]. Current research indicates that epigenetic factors directly or indirectly regulate various abiotic stresses, including drought [65], salinity [66], high temperatures [67], freezing damage [68], and ultraviolet radiation [69]. In our investigations of epigenetic modifications in the two cultivars under flooding stress, we observed a downregulation of both *MET1* and *DME* in flooding-sensitive ND45. *MET1* is involved in maintaining DNA methylation, and reduced *MET1* levels may lead to decreased stability of DNA methylation, resulting in increased gene expression variability [70]. Meanwhile, *DME* promotes the demethylation process by facilitating the removal of methyl groups from DNA. When *DME* is downregulated, this demethylation process is inhibited [71]. *MET1* and *DME* play crucial roles in plants, especially when facing biotic or abiotic stresses [72,73]. The simultaneous downregulation of both *MET1* and *DME* presents a seemingly contradictory scenario. This complex interplay where the maintenance of DNA methylation is impaired, while demethylation processes are inhibited, signifies an unstable epigenetic environment that can significantly influence gene expression in response to flooding stress in the flooding-sensitive cultivar ND45. Future research should focus on elucidating the mechanisms underlying the interplay between *MET1* and *DME*, as well as their impacts on gene expression profiles, to enhance our understanding of flood tolerance in soybeans and inform breeding strategies for improved resilience against abiotic stresses.

In addition, plant flooding tolerance entails a complex interplay that confer tolerance to adverse environmental conditions, with various signaling pathways playing pivotal roles [74]. Under flooding stress, plants encounter challenges related to the transportation of oxygen, water balance, and energy supply, necessitating adaptive mechanisms for survival. For example, GLB proteins have been reported to participate in oxygen transport under water stress, thereby promoting plant growth and lateral root elongation [63,75]. Channel proteins, such as AQPs, can promote the transportation of water and other solutes across cell membranes, maintaining normal cellular processes [76,77]. Additionally, energy requirements are met through pathways such as glycolysis/gluconeogenesis and alcohol fermentation [62,78]. In our study, the signaling molecules of these pathways were analyzed through q-PCR. It was found that the expression pattern changes of most genes in these pathways were consistent between ND38 and ND45 during flooding treatment, such as *GLB1-1*, *GLB1-2*, and *GLB2-2* related to oxygen transport; *PIP2-6*, *PIP2-7*, *TIP2-2*, *TIP4-1*, and *TIP5-1* involved in water balance; and *Gm02G222400*, *Gm19G017200*, and *Gm04G213900* associated with energy metabolism. These results are consistent with previous reports highlighting the involvement of these genes in response to flooding stress, suggesting their functional conservation across various species. It also indicates that there are multiple signaling pathways involved when soybeans respond to flooding stress.

Conventional breeding has long been considered the cornerstone method for developing superior varieties, leveraging techniques like hybridization, introduction, and backcrossing to imbue desirable agronomic traits [63]. The quest for cultivars harboring advantageous traits has remained a primary aspiration for breeders. Over years of dedicated exploration, several soybean cultivars with flooding tolerance have been developed, including NN1138-2, M8206, ZXD, TGX 1990-94F, SBO-115, GC-840, BINAsoybean1, BARISoybean5, Sohag, and BINAsoybean2 [64,79,80]. These varieties stand as valuable reservoirs of genetic diversity, offering promising foundations for future flooding-tolerance breeding endeavors. In our research, through the comparison between the flooding-tolerant ND38 and flooding-sensitive ND45, we were able to identify key genetic and physiological factors contributing to the differential responses of soybeans to flooding stress, shedding light on potential targets for breeding resilient cultivars.

## 5. Conclusions

This study highlights the distinct physiological and molecular responses of two soybean cultivars, ND38 and ND45, to flooding stress, demonstrating the critical need for developing flooding-tolerant varieties. ND38 exhibited superior tolerance compared with ND45, as evidenced by reduced plant height, root length, and chlorophyll content under flooding conditions. Our investigation revealed that various pathways—including those related to glycolysis/gluconeogenesis, hemoglobin, aquaporins, and epigenetic modifications—play significant roles in these adaptive responses. Importantly, the differential expression of key genes related to oxygen transport and energy metabolism indicated a complex interplay that facilitates survival under flooding stress. Our research underscores the importance of understanding these mechanisms to inform breeding strategies aimed at enhancing soybean tolerance in particularly flood-prone regions.

## Figures and Tables

**Table 1:** Primer sequences of qPCR.

Gene ID	Forward Primer Sequence	Reverse Primer Sequence	Reference
GmDMEs	AATCCAACTGGGCATCCAGG	TGAACTTGGGGCTTGTGTGT	[43]
GmMET1	ACGCTGAGAAGACAACCACA	CACTTGCAGTTGCATGGGTC	[44]
GmNAC11	TTGCCACCCGGTTTTAGGTT	CTGCAATGATGGAAACGGGC	[45]
GmNAC61	CCTCAGAAGGTTCCAAGTGC	TCCTGCAGATAGCCCAAGAT	
GmNAC124	TCCATACCCTTGACCGTTTC	CCTTGCACCATTTGGGTACT	
GmNAC151	TCTGTCGAAGCTGAAGACGA	TGGTCTTCCAGTAGCCCCCTA	
GmPIP2-6	CTGGAACCGGCATTAACCCT	GCTCCAACAAACGGTCCAAC	[46]
GmPIP2-7	TTTCTGGCGAGGAAGGTGTC	CCAAACCGGTTCCTTTGCTG	
GmROS1	ACCATCAAAGAACGGGGCAT	TGCAGTGTTAAAAGCCGCAC	[47]
GmTIP2-2	TTTGTAGGTGTCTCCGTCGC	ATGCTCTTGGCGGTGATGAA	[48]
GmTIP4-1	CATCTTTCGTTCCCTGCTCT	AGTTGCCTGTCCTCCAGAGA	
GmTIP5-1	CCTCACGGAAGTTGATGCCT	CCATTGCAAAGGTCACAGCC	
Gm02G222400	TGGGAAGAAGCCATGGTCAC	TCTGAGGCACCATCAGCAAG	[49]
Gm04G213900	AGCTGGAAAGCCATTGGTGA	AAAGGTGTCTGTCCCTTGGC	
Gm08G165400	ACGTGAATGATGCTTTCGGC	GAATCCTGCAACAGAGGGCT	
Gm18G219100	GTGGAGATTGGTCAGGGCTC	AAATATGCGACCAGCCCCAA	
Gm19G000700	AGTCCATTGGAGAGCCTTGC	CTTAGCTGTAGACCCGCCAC	
Gm19G017200	CAACCAGATGCCCTTGCCTA	GAAGGTCGGTTGCCTGAGAA	
GmGLB1-1	AGCAGTGCCTGAAATGTGGT	GATTTGATGGCTTCGGCCAG	[29]
GmGLB1-2	CCATGCCGTGTCTGTCTTTG	ACGCCGGTTCTAAAATGGGT	
GmGLB2-1	CCGCACTTGGTTCTATCCAT	GCTGCTGCCAATTCATCATA	
GmGLB2-2	TGATGCCACACTTGGTCCTA	GCCATTGCCTTCTTAATTGC	
GmGLB2-3	ACCTGCAGCAAAGGACTTGT	GCTTTTTGGGCATGGATAGA	
GmACT11	GGTGGTTCTATCTTGGCATC	CTTTCGCTTCAATAACCCTA	/

## Data Availability

The original contributions presented in this study are included in the article/Supplementary Material, further inquiries can be directed to the corresponding authors.

## Supplementary Materials

The following supporting information can be downloaded at: https://www.mdpi.com/article/10.3390/cimb46110739/s1, Table S1: Growth parameters of soybean seedlings; Table S2: Chlorophyll content analysis of soybean leaves.

## References

[1] Pazdernik D.L., Killam A.S., Orf J.H. (1997). Analysis of amino and fatty acid composition in soybean seed, using near infrared reflectance spectroscopy. Agron. J..

[2] Yin Y., Fatufe A.A., Blachier F., Blachier F. (2011). Soya bean meal and its extensive use in livestock feeding and nutrition. Soybean and Nutrition.

[3] Liu S., Zhang M., Feng F., Tian Z. (2020). Toward a “Green Revolution” for Soybean. Mol. Plant.

[4] Nguyen C.X., Paddock K.J., Zhang Z., Stacey M.G. (2021). GmKIX8-1 regulates organ size in soybean and is the causative gene for the major seed weight QTL qSw17-1. New Phytol..

[5] Bisht A., Saini D.K., Kaur B., Batra R., Kaur S., Kaur I., Jindal S., Malik P., Sandhu P.K., Kaur A. (2023). Multi-omics assisted breeding for biotic stress resistance in soybean. Mol. Biol. Rep..

[6] Tang Y., Lu S., Fang C., Liu H., Dong L., Li H., Su T., Li S., Wang L., Cheng Q. (2023). Diverse flowering responses subjecting to ambient high temperature in soybean under short-day conditions. Plant Biotechnol. J..

[7] Li N., Nie T., Tang Y., Lu D., Wang T., Zhang Z., Chen P., Li T., Meng L., Jiao Y. (2022). Responses of Soybean Water Supply and Requirement to Future Climate Conditions in Heilongjiang Province. Agriculture.

[8] Wang Q., Ning Z., Awan S.A., Gao J., Chen J., Lei Y., Tan X., Wu X., Wu Y., Liu C. (2023). Far-Red light mediates light energy capture and distribution in soybeans (*Glycine max* L.) under the shade. Plant Physiol. Biochem..

[9] Liu M., Linna C., Ma S., Ma Q., Guo J., Wang F., Wang L. (2022). Effects of biochar with inorganic and organic fertilizers on agronomic traits and nutrient absorption of soybean and fertility and microbes in purple soil. Front. Plant Sci..

[10] Jumrani K., Bhatia V.S. (2018). Impact of combined stress of high temperature and water deficit on growth and seed yield of soybean. Physiol. Mol. Biol. Plants.

[11] Staniak M., Szpunar-Krok E., Kocira A. (2023). Responses of Soybean to Selected Abiotic Stresses—Photoperiod, Temperature and Water. Agriculture.

[12] Bagale S., Abdelhamid M. (2021). Nutrient Management for Soybean Crops. Int. J. Agron..

[13] Eulenstein F., Lana M., Schlindwein S., Sheudzhen A., Tauschke M., Behrend A., Guevara E., Meira S. (2016). Trends of soybean yields under climate change scenarios. Horticulturae.

[14] Zong C., Zhao J., Wang Y., Wang L., Chen Z., Qi Y., Bai Y., Li W., Wang W., Ren H. (2024). Identification of Gene–Allele System Conferring Alkali-Tolerance at Seedling Stage in Northeast China Soybean Germplasm. Int. J. Mol. Sci..

[15] Lu L., Wei W., Tao J.J., Lu X., Bian X.H., Hu Y., Cheng T., Yin C.C., Zhang W.K., Chen S.Y. (2021). Nuclear factor Y subunit GmNFYA competes with GmHDA13 for interaction with GmFVE to positively regulate salt tolerance in soybean. Plant Biotechnol. J..

[16] Rasheed A., Mahmood A., Maqbool R., Albaqami M., Sher A., Sattar A., Bakhsh G., Nawaz M., Hassan M.U., Al-Yahyai R. (2022). Key insights to develop drought-resilient soybean: A review. J. King Saud. Univ.-Sci..

[17] Wang X., Komatsu S. (2020). Proteomic techniques for the development of flood-tolerant soybean. Int. J. Mol. Sci..

[18] Jia W., Ma M., Chen J., Wu S. (2021). Plant morphological, physiological and anatomical adaption to flooding stress and the underlying molecular mechanisms. Int. J. Mol. Sci..

[19] Liu J., Li X., Li Y., Sirisrisakulchai J., Kang X., Cui J. (2024). Decomposition and Driving Factors of Total Factor Productivity of Food Crops in the Yellow River Basin, China. Agriculture.

[20] Li T., Li J., Zhang D.D. (2020). Yellow River flooding during the past two millennia from historical documents. Prog. Phys. Geogr. Earth Environ..

[21] Wei K., Ouyang C., Duan H., Li Y., Chen M., Ma J., An H., Zhou S. (2020). Reflections on the catastrophic 2020 Yangtze River Basin flooding in southern China. Innovation.

[22] Khan M.N., Ahmed I., Ud Din I., Noureldeen A., Darwish H., Khan M. (2022). Proteomic insight into soybean response to flooding stress reveals changes in energy metabolism and cell wall modifications. PLoS ONE.

[23] Kigel J. (2017). Seed germination in arid and semiarid regions. Seed Development and Germination.

[24] Kabrick J.M., Dey D.C., Van Sambeek J., Coggeshall M.V., Jacobs D.F. (2012). Quantifying flooding effects on hardwood seedling survival and growth for bottomland restoration. New For..

[25] Purcell L.C., Salmeron M., Ashlock L. (2014). Soybean growth and development. Arkansas Soybean Production Handbook.

[26] Mishra N., Jiang C., Chen L., Paul A., Chatterjee A., Shen G. (2023). Achieving abiotic stress tolerance in plants through antioxidative defense mechanisms. Front. Plant Sci..

[27] Du H., Shen X., Huang Y., Huang M., Zhang Z. (2016). Overexpression of Vitreoscilla hemoglobin increases waterlogging tolerance in Arabidopsis and maize. BMC Plant Biol..

[28] Shi X., Wang X., Peng F., Zhao Y. (2012). Molecular cloning and characterization of a nonsymbiotic hemoglobin gene (GLB1) from Malus hupehensis Rehd. with heterologous expression in tomato. Mol. Biol. Rep..

[29] Koltun A., Fuhrmann-Aoyagi M.B., Cardoso Moraes L.A., Lima Nepomuceno A., Simoes Azeredo Goncalves L., Mertz-Henning L.M. (2022). Uncovering the roles of hemoglobins in soybean facing water stress. Gene.

[30] Li Y., Zhang W., Zhu W., Zhang B., Huang Q., Su X. (2020). Waterlogging tolerance and wood properties of transgenic Populus alba × glandulosa expressing *Vitreoscilla hemoglobin* gene (Vgb). J. For. Res..

[31] Reddy P.S., Rao T.S.R.B., Sharma K.K., Vadez V. (2015). Genome-wide identification and characterization of the aquaporin gene family in *Sorghum bicolor* (L.). Plant Gene.

[32] Cheng X.-F., Wu H.-H., Zou Y.-N., Wu Q.-S., Kuča K. (2021). Mycorrhizal response strategies of trifoliate orange under well-watered, salt stress, and waterlogging stress by regulating leaf aquaporin expression. Plant Physiol. Biochem..

[33] Castonguay Y., Nadeau P., Simard R. (1993). Effects of flooding on carbohydrate and ABA levels in roots and shoots of alfalfa. Plant Cell Environ..

[34] Wang X., Zhu W., Hashiguchi A., Nishimura M., Tian J., Komatsu S. (2017). Metabolic profiles of flooding-tolerant mechanism in early-stage soybean responding to initial stress. Plant Mol. Biol..

[35] Zeng R., Chen T., Wang X., Cao J., Li X., Xu X., Chen L., Xia Q., Dong Y., Huang L. (2021). Physiological and Expressional Regulation on Photosynthesis, Starch and Sucrose Metabolism Response to Waterlogging Stress in Peanut. Front. Plant Sci..

[36] Borrego-Benjumea A., Carter A., Tucker J.R., Yao Z., Xu W., Badea A. (2020). Genome-Wide Analysis of Gene Expression Provides New Insights into Waterlogging Responses in Barley (*Hordeum vulgare* L.). Plants.

[37] Rauf M., Arif M., Fisahn J., Xue G.P., Balazadeh S., Mueller-Roeber B. (2013). NAC transcription factor speedy hyponastic growth regulates flooding-induced leaf movement in Arabidopsis. Plant Cell.

[38] Li M., Zhang Y., Li D., Wang Y., Zhou R., Wang L., Zhang Y., Yu J., Gong H., You J. (2018). Genome-wide identification and comprehensive analysis of the NAC transcription factor family in *Sesamum indicum*. PLoS ONE.

[39] Venkategowda R., Cao M., Zheng L., Li J., Mao Y., Zhang R., Niu X., Geng M., Zhang X., Huang W. (2022). Transcriptomic profiling suggests candidate molecular responses to waterlogging in cassava. PLoS ONE.

[40] Bhanu B.D., Alluri A., Shanker A.K., Ulaganathan K. (2022). DNA methylation in plants and its role in abiotic stress tolerance. Climate Change and Crop Stress Molecules to Ecosystems.

[41] Pan R., Xu Y.H., Xu L., Zhou M.X., Jiang W., Wang Q., Zhang W.Y. (2020). Methylation Changes in Response to Hypoxic Stress in Wheat Regulated by Methyltransferases. Russ. J. Plant Physiol..

[42] Dossa K., Mmadi M.A., Zhou R., Zhou Q., Yang M., Cisse N., Diouf D., Wang L.H., Zhang X.R. (2018). The contrasting response to drought and waterlogging is underpinned by divergent DNA methylation programs associated with transcript accumulation in sesame. Plant Sci..

[43] Wang W., Zhang T., Liu C., Liu C., Jiang Z., Zhang Z., Ali S., Li Z., Wang J., Sun S. (2024). A DNA demethylase reduces seed size by decreasing the DNA methylation of AT-rich transposable elements in soybean. Commun. Biol..

[44] Coelho F.S., Miranda S.S., Moraes J.L., Hemerly A.S., Ballesteros H.G.F., Santa-Catarina C., Dos Santos R.C., de Almeida F.A., Silveira V., Macedo A. (2024). DNA methylation impacts soybean early development by modulating hormones and metabolic pathways. Physiol. Plant..

[45] Pinheiro G.L., Marques C.S., Costa M.D., Reis P.A., Alves M.S., Carvalho C.M., Fietto L.G., Fontes E.P. (2009). Complete inventory of soybean NAC transcription factors: Sequence conservation and expression analysis uncover their distinct roles in stress response. Gene.

[46] Fleurat-Lessard P., Michonneau P., Maeshima M., Drevon J.-J., Serraj R. (2005). The distribution of aquaporin subtypes (PIP1, PIP2 and γ-TIP) is tissue dependent in soybean (*Glycine max*) root nodules. Ann. Bot..

[47] Liang X., Hou X., Li J., Han Y., Zhang Y., Feng N., Du J., Zhang W., Zheng D., Fang S. (2019). High-resolution DNA methylome reveals that demethylation enhances adaptability to continuous cropping comprehensive stress in soybean. BMC Plant Biol..

[48] Feng Z.-J., Liu N., Zhang G.-W., Niu F.-G., Xu S.-C., Gong Y.-M. (2019). Investigation of the AQP family in soybean and the promoter activity of TIP2; 6 in heat stress and hormone responses. Int. J. Mol. Sci..

[49] Lin Y., Li W., Zhang Y., Xia C., Liu Y., Wang C., Xu R., Zhang L. (2019). Identification of genes/proteins related to submergence tolerance by transcriptome and proteome analyses in soybean. Sci. Rep..

[50] Tian L.-X., Zhang Y.-C., Chen P.-L., Zhang F.-F., Li J., Yan F., Dong Y., Feng B.-L. (2021). How Does the Waterlogging Regime Affect Crop Yield? A Global Meta-Analysis. Front. Plant Sci..

[51] Ploschuk R.A., Miralles D.J., Colmer T.D., Ploschuk E.L., Striker G.G. (2018). Waterlogging of Winter Crops at Early and Late Stages: Impacts on Leaf Physiology, Growth and Yield. Front. Plant Sci..

[52] de San Celedonio R.P., Abeledo L.G., Miralles D.J. (2014). Identifying the critical period for waterlogging on yield and its components in wheat and barley. Plant Soil..

[53] Ma S., Hou J., Wang Y., Wang M., Zhang W., Fan Y., Huang Z. (2022). Post-flowering Soil Waterlogging Curtails Grain Yield Formation by Restricting Assimilates Supplies to Developing Grains. Front. Plant Sci..

[54] Zhang X., Huang C., Meng Y., Liu X., Gao Y., Liu Z., Ma S. (2023). Physiological Mechanism of Waterlogging Stress on Yield of Waxy Maize at the Jointing Stage. Plants.

[55] Yin D., Sun D., Han Z., Ni D., Norris A., Jiang C.-Z. (2019). PhERF2, an ethylene-responsive element binding factor, plays an essential role in waterlogging tolerance of petunia. Hortic. Res..

[56] Hattori Y., Nagai K., Furukawa S., Song X.-J., Kawano R., Sakakibara H., Wu J., Matsumoto T., Yoshimura A., Kitano H. (2009). The ethylene response factors SNORKEL1 and SNORKEL2 allow rice to adapt to deep water. Nature.

[57] Xu K., Xu X., Fukao T., Canlas P., Maghirang-Rodriguez R., Heuer S., Ismail A.M., Bailey-Serres J., Ronald P.C., Mackill D.J. (2006). Sub1A is an ethylene-response-factor-like gene that confers submergence tolerance to rice. Nature.

[58] Yu F., Liang K., Fang T., Zhao H., Han X., Cai M., Qiu F. (2019). A group VII ethylene response factor gene, ZmEREB180, coordinates waterlogging tolerance in maize seedlings. Plant Biotechnol. J..

[59] Komatsu S., Thibaut D., Hiraga S., Kato M., Chiba M., Hashiguchi A., Tougou M., Shimamura S., Yasue H. (2011). Characterization of a novel flooding stress-responsive alcohol dehydrogenase expressed in soybean roots. Plant Mol. Biol..

[60] Song L., Valliyodan B., Prince S., Wan J., Nguyen H. (2018). Characterization of the XTH Gene Family: New Insight to the Roles in Soybean Flooding Tolerance. Int. J. Mol. Sci..

[61] Oliveira F.K.d., Da-Silva C.J., Garcia N., Agualongo D.A.P., de Oliveira A.C.B., Kanamori N., Takasaki H., Urano K., Shinozaki K., Nakashima K. (2022). The overexpression of NCED results in waterlogging sensitivity in soybean. Plant Stress..

[62] Pan J., Sharif R., Xu X., Chen X. (2020). Mechanisms of Waterlogging Tolerance in Plants: Research Progress and Prospects. Front. Plant Sci..

[63] Yijun G., Zhiming X., Jianing G., Qian Z., Rasheed A., Hussain M.I., Ali I., Shuheng Z., Hassan M.U., Hashem M. (2022). The intervention of classical and molecular breeding approaches to enhance flooding stress tolerance in soybean—An review. Front. Plant Sci..

[64] Sathi K.S., Masud A.A.C., Falguni M.R., Ahmed N., Rahman K., Hasanuzzaman M., Nikalje G. (2022). Screening of Soybean Genotypes for Waterlogging Stress Tolerance and Understanding the Physiological Mechanisms. Adv. Agric..

[65] Banerjee A., Roychoudhury A. (2017). Epigenetic regulation during salinity and drought stress in plants: Histone modifications and DNA methylation. Plant Gene.

[66] Singroha G., Kumar S., Gupta O.P., Singh G.P., Sharma P. (2022). Uncovering the epigenetic marks involved in mediating salt stress tolerance in plants. Front. Genet..

[67] Liu J., Feng L., Li J., He Z. (2015). Genetic and epigenetic control of plant heat responses. Front. Plant Sci..

[68] Satyakam, Zinta G., Singh R.K., Kumar R. (2022). Cold adaptation strategies in plants—An emerging role of epigenetics and antifreeze proteins to engineer cold resilient plants. Front. Genet..

[69] Molinier J. (2017). Genome and epigenome surveillance processes underlying UV exposure in plants. Genes.

[70] Zhang H., Lang Z., Zhu J.-K. (2018). Dynamics and function of DNA methylation in plants. Nat. Rev. Mol. Cell Biol..

[71] Li Y., Kumar S., Qian W. (2018). Active DNA demethylation: Mechanism and role in plant development. Plant Cell Rep..

[72] Bhattarai K., Poudel M.R. (2022). DNA methylation and plants response to biotic and abiotic stress. Trends Sci..

[73] Roldán-Arjona T., Ariza R.R. (2009). DNA demethylation. DNA and RNA Modification Enzymes: Structure, Mechanisms, Functions and Evolution.

[74] De Oliveira M.R., Wu C., Harrison D., Florez-Palacios L., Acuna A., Da Silva M.P., Ravelombola S.F., Winter J., Rupe J., Shakiba E. (2022). Response to selection to different breeding methods for soybean flood tolerance. Crop Sci..

[75] Becana M., Yruela I., Sarath G., Catalán P., Hargrove M.S. (2020). Plant hemoglobins: A journey from unicellular green algae to vascular plants. New Phytol..

[76] Song L., Nguyen N., Deshmukh R.K., Patil G.B., Prince S.J., Valliyodan B., Mutava R., Pike S.M., Gassmann W., Nguyen H.T. (2016). Soybean TIP Gene Family Analysis and Characterization of GmTIP1;5 and GmTIP2;5 Water Transport Activity. Front. Plant Sci..

[77] Tan X., Xu H., Khan S., Equiza M.A., Lee S.H., Vaziriyeganeh M., Zwiazek J.J. (2018). Plant water transport and aquaporins in oxygen-deprived environments. J. Plant Physiol..

[78] Ren C.-G., Kong C.-C., Yan K., Zhang H., Luo Y.-M., Xie Z.-H. (2017). Elucidation of the molecular responses to waterlogging in Sesbania cannabina roots by transcriptome profiling. Sci. Rep..

[79] Ali M.J., Yu Z., Xing G., Zhao T., Gai J. (2017). Establishment of evaluation procedure for soybean seed-flooding tolerance and its application to screening for tolerant germplasm sources. Legume Res.-Int. J..

[80] Pokhrel A., Shrestha R., Dangi S.R. (2021). Screening of Soybean Genotypes to Short Period of Flooding. Agron. J. Nepal..

