# Supplementary material for: Comparative Analysis of Two Soybean Cultivars Revealed Tolerance Mechanisms Underlying Soybean Adaptation to Flooding
Source: Curr Issues Mol Biol. 2024 Nov 4;46(11):12442–56. doi: 10.3390/cimb46110739 (PMC11592816; doi:10.3390/cimb46110739)
Supplement: Supplementary file 1 [file cimb-46-00739-s001.zip › cimb-3248113-supplementary.pdf]

Table S1 Phenotype parameters

	ND38		Significance	P values	ND45		Significance	P values
	CK	Flooding			CK	Flooding		
Plant height of seedlings (cm)	23	14	p<0.05 *	p=0.013	27	20	p<0.05 *	p=0.034
	19	13			26	19		
	21	15			23	19		
ength of seedling roots (cm)	41	20	ns	p=0.182	51	22	ns	p=0.125
	23	17			32	24		
	25	18			33	23		
Dry weight of aboveground parts (g)	0.546	0.601	ns	p=0.447	0.882	1.052	ns	p=0.0828
	0.687	0.395			0.78	1.009		
	0.444	0.462			0.945	0.962		
Dry weight of underground parts (g)	0.282	0.143	p<0.001 ***	p=7.35e-09	0.236	0.252	p<0.001 ***	p=4e-05

Table S2 Chlorophyll Values

	ND38		Significance	P values	ND45		Significance	P values
	CK	Flooding			CK	Flooding		
Total chlorophyll content (%)	67.19	46.459	P<0.01 **	p=0.00852	86.16	47.486	P<0.01 **	p=0.0032
	72.61	40.312			85.29	55.634		
	81.96	52.273			83.72	48.735		
Chlorophyll a content (mg/mL)	0.029	0.024	ns	p=0.104	0.029	0.025	ns	p=0.134
	0.028	0.022			0.028	0.028		
	0.028	0.027			0.028	0.025		
Chlorophyll b content (mg/mL)	0.546	0.601	P<0.01 **	p=0.0115	0.882	1.052	P<0.01 **	p=0.0032
	0.687	0.395			0.78	1.009		
	0.444	0.462			0.945	0.962		
